# Exosomes play a role in multiple myeloma bone disease and tumor development by targeting osteoclasts and osteoblasts

**DOI:** 10.1038/s41408-018-0139-7

**Published:** 2018-11-08

**Authors:** Sylvia Faict, Joséphine Muller, Kim De Veirman, Elke De Bruyne, Ken Maes, Louise Vrancken, Roy Heusschen, Hendrik De Raeve, Rik Schots, Karin Vanderkerken, Jo Caers, Eline Menu

**Affiliations:** 1 Department of Hematology and Immunology, Myeloma Center Brussels, Vrije Universiteit Brussel, Brussels, Belgium; 2 Laboratory of Hematology, GIGA-Research, University of Liège, Liège, Belgium; 3 Department of Pathology, UZ Brussel, Vrije Universiteit Brussel, Brussels, Belgium; 4 Department of Clinical Haematology, Universitair Ziekenhuis Brussel, Brussels, Belgium; 5 Division of Hematology, Department of Medicine, University and CHU of Liège, Liège, Belgium

## Abstract

Progression of multiple myeloma (MM) is largely dependent on the bone marrow (BM) microenvironment wherein communication through different factors including extracellular vesicles takes place. This cross-talk not only leads to drug resistance but also to the development of osteolysis. Targeting vesicle secretion could therefore simultaneously ameliorate drug response and bone disease. In this paper, we examined the effects of MM exosomes on different aspects of osteolysis using the 5TGM1 murine model. We found that 5TGM1 sEVs, or ‘exosomes’, not only enhanced osteoclast activity, they also blocked osteoblast differentiation and functionality in vitro. Mechanistically, we could demonstrate that transfer of DKK-1 led to a reduction in Runx2, Osterix, and Collagen 1A1 in osteoblasts. In vivo, we uncovered that 5TGM1 exosomes could induce osteolysis in a similar pattern as the MM cells themselves. Blocking exosome secretion using the sphingomyelinase inhibitor GW4869 not only increased cortical bone volume, but also it sensitized the myeloma cells to bortezomib, leading to a strong anti-tumor response when GW4869 and bortezomib were combined. Altogether, our results indicate an important role for exosomes in the BM microenvironment and suggest a novel therapeutic target for anti-myeloma therapy.

## Introduction

Osteolysis is one of the main hallmarks of multiple myeloma (MM) disease and results from a disrupted homeostasis in bone formation and resorption. At the time of diagnosis, osteolytic lesions are present in 60% of patients. In addition, almost every patient will manifest a lytic lesion at some point during their disease course, resulting in increased morbidity and pain with ultimately a severe impact on the quality of life^1–3^.

MM is caused by a clonal expansion of plasma cells in the bone marrow (BM) where malignant cells interact with their microenvironment to create a protective niche^4^. Our group and others have examined the implication of exosomes in the cross-talk between MM cells and the microenvironment. Exosomes are a subfraction of extracellular vesicles (EVs), ranging in size from 35–120 nm. They are actively secreted; contain cell-specific, bioactive molecules and exert their functions by transferring their cargo to the target cells, either by endocytosis or by direct fusion with the cell membrane^5^. We have previously demonstrated that exosomes from BMSCs and myeloma cells enhance MM progression by the induction of drug resistance, angiogenesis, and immune suppression^6–10^.

So far, only a few papers have studied the role of EVs in MM osteolysis, thereby focusing on osteoclast activation^11,12^. A recent study suggests a role for IL-32 positive EVs secreted in hypoxia by certain MM cells^11^. Herein IL-32 induced the nuclear translocation of NF-kB, leading to the stimulation of osteoclastic differentiation and activation. Bone resorption is further accelerated by inhibition of bone formation. This is triggered by the release of molecules such as DKK-1 and sFRP2, both inhibitors of the Wnt pathway, which ultimately lead to a block in osteoblast proliferation and differentiation^1^. To our knowledge, no studies have looked at the effect of MM EVs on osteoblast functionality nor at the effect of inhibiting exosome secretion in the MM microenvironment in a relevant mouse model.

The treatment, and if possible, prevention, of multiple myeloma bone disease (MMBD) must be a priority in MM treatment. Until today, the standard treatment of MMBD mainly focuses on the inhibition of osteoclasts by administering bisphosphonates^1^. However, these bisphosphonates do not have any direct effect on bone formation, but rather inhibit the general bone turnover. Therefore, there is an urgent need to find novel targets that would not only treat osteolysis, but also reduce tumor growth directly and indirectly by interfering in the BM microenvironment. Since exosomes play such a prominent role in the MM microenvironment, they could represent this novel target.

In this paper, we first established the osteolytic effects of MM small EVs (sEVs) or exosomes, in vivo. Next, we evaluated the effect of these sEVs in vitro, both on osteoblasts as well as on osteoclasts. Finally, we analyzed the effect of blocking exosome secretion in vivo on osteolysis and overall tumor burden, when combined with the standard treatment bortezomib.

## Materials and methods

### Mice and cell lines

C57BL/KalwRij mice were purchased from Envigo Laboratories, Horst, The Netherlands. They were housed and treated following conditions approved by the Ethical Committee for Animal Experiments of the Vrije Universiteit Brussel (licence No LA1230281, CEP No 15-281-3). Used cell lines and culture conditions are described in supplementary methods.

### Drugs and reagents

Bortezomib was purchased from Selleckchem (Munich, Germany) and GW4869 was purchased from Sigma-Aldrich (St. Louis, MO, USA). Both were dissolved in dimethylsulfoxide according to manufacturer’s instructions. For in vivo use, both were further diluted in PBS to their appropriate concentration.

### Osteoblast differentiation

MC3T3-E1 cells were seeded at a density of 25,000 cells/cm^2^ and grown to 70–80% confluence. Osteoblast differentiation was initiated by changing the culture medium to αMEM supplemented with 10% FCS, 2 mM L-glutamine, 1% P/S, 50 μg/ml ascorbic acid and 3 mM β-glycerophosphate (Sigma-Aldrich). This osteogenic medium was refreshed twice per week. Experiments with differentiated MC3T3-E1 cells were performed on day 14 after the start of differentiation.

### Osteoclast differentiation, TRAP staining, and resorptive activity

Osteoclast differentiation and resorptive activity were studied as previously described^13,14^. Briefly, RAW264.7 cells were initially seeded in αMEM medium supplemented with recombinant murine sRANKL (Peprotech, London, UK) at day 1. Medium was refreshed after 3 days, with the addition of either 5TGM1 concentrated conditioned medium (CCM), 5TGM1 sEVs (100 µg/ml) or sRANKL. 1 day later, the osteoclast cultures were stopped, cells were fixed in 4% paraformaldehyde and stained for TRAP activity using the Leukocyte Tartrate-Resistant Acid Phosphatase kit (Sigma-Aldrich). To assess the resorptive capacity of osteoclasts, RAW264.7 cells were seeded on Osteo Assay 96-well plates (Corning, New York, USA) in osteoclast differentiation medium. The medium was refreshed every 3 days. After 12 days, a Von Kossa staining was performed to visualize non-resorbed matrix. The number of resorption pits and average pit size were quantified using ImageJ software (NIH, Bethesda, USA).

### Isolation and characterization of small EVs

5TGM1 cells were cultured without serum for 24 h and conditioned medium (CM) was collected after centrifugation and filtered using a 0.22 µM pore filter (Carl Roth, Karlsruhe, Germany). The filtered medium was concentrated using a 150 kD Protein Concentrator (Thermo Scientific, Waltham, MA, USA) and filtered again with a 0.22 µM pore filter.

From this concentrated CM (CCM), small EVs were isolated using ExoQuick-TC exosome precipitation solution (System biosciences, Mountain View, CA, USA) according to manufacturer’s instructions, with the addition of an extra filter and a final high speed centrifugation step (10,000 × *g*, 2 min) to eliminate contaminating cell debris, proteins and larger EVs.

Results of experiments with Exoquick-EVs were confirmed with sEVs isolated by Optiprep Density gradient Ultracentrifugation, as described in supplementary methods.

The concentration of EV proteins was determined by BCA protein analysis (Pierce BCA protein Assay, Thermo Scientific). Size and number of EVs was determined by the Zetaview (Distrilab, Leusden, Netherlands), with the average size and number calculated from 11 independent replicates using nanoparticle tracking analysis.

### In vivo administration of exosomes

5-week old naive C57BL6/KalwRij female mice were intravenously injected with 200 μg of 5TGM1 exosomes three times per week. As a positive control, we simultaneously injected 1 million 5TGM1 cells intravenously once, at day 0. After 3 weeks, mice were sacrificed and one femur per mouse was defleshed and stored at 70% EtOH for micro-computed tomography (µCT) analysis. Spleen was isolated and weighed. One tibia was flushed and plasma cell count in the BM was determined after cytosmear staining with May–Grünwald Giemsa to ensure there was no myeloma present.

### Alkaline phosphatase (ALP) activity

MC3T3-E1 were seeded in a 96-well plate and cultured in either no serum medium, 5TGM1 CCM or no serum medium containing 5TGM1 sEVs (100 µg/ml). After 24 h, medium was removed and cells were lysed using lysis buffer. Protein concentration was measured by BCA analysis (Pierce BCA protein Assay, Thermo Scientific), and ALP activity was measured using the Alkaline Phosphatase Yellow Liquid substrate for ELISA (P7998, Sigma-Aldrich) according to manufacturer’s instructions. Absorbance was read at 415 nm with the BioRad iMark™ Microplate Absorbance reader.

### In vivo treatment with GW4869 and bortezomib

6-week old C57BL6/KalwRij mice were inoculated intravenously with 1 million 5TGM1 cells. Mice were treated from day +1 with GW4869 (2.5 mpk) intraperitoneally three times per week and/or bortezomib (0.6 mpk) subcutaneously two times per week. When first mice showed sign of hind-limb paralysis, all mice were sacrificed. Percentage of 5TGM1 cells in the BM was counted after cytosmear staining with May–Grünwald Giemsa. M-protein in serum was determined by protein electrophoresis. One femur was defleshed and stored in 70% EtOH for µCT analysis. The contralateral femur was used for quantification of angiogenesis, as described in supplementary methods.

### Statistical analysis

Results were analyzed with GraphPad Prism 5.0 software (GraphPad Software Inc, La Jolla, CA, USA). All data represent the mean ± standard deviation (SD), and results were analyzed using the Mann–Whitney *U* non-parametric test and One-way ANOVA for multiple testing. *p* < 0.05 (*), *p* < 0.01 (**) and *p* < 0.001 (***) were considered statistically significant.

Details about the collection of conditioned medium, western blot analysis, viability assays, RT-PCR analysis, transmission electron microscopy, µCT, Collagen type I degradation product ELISA, fluorescent labeling, and confocal microscopy are included in supplementary materials.

## Results

### Characterization of 5TGM1 extracellular vesicles

**Fig. 1:**
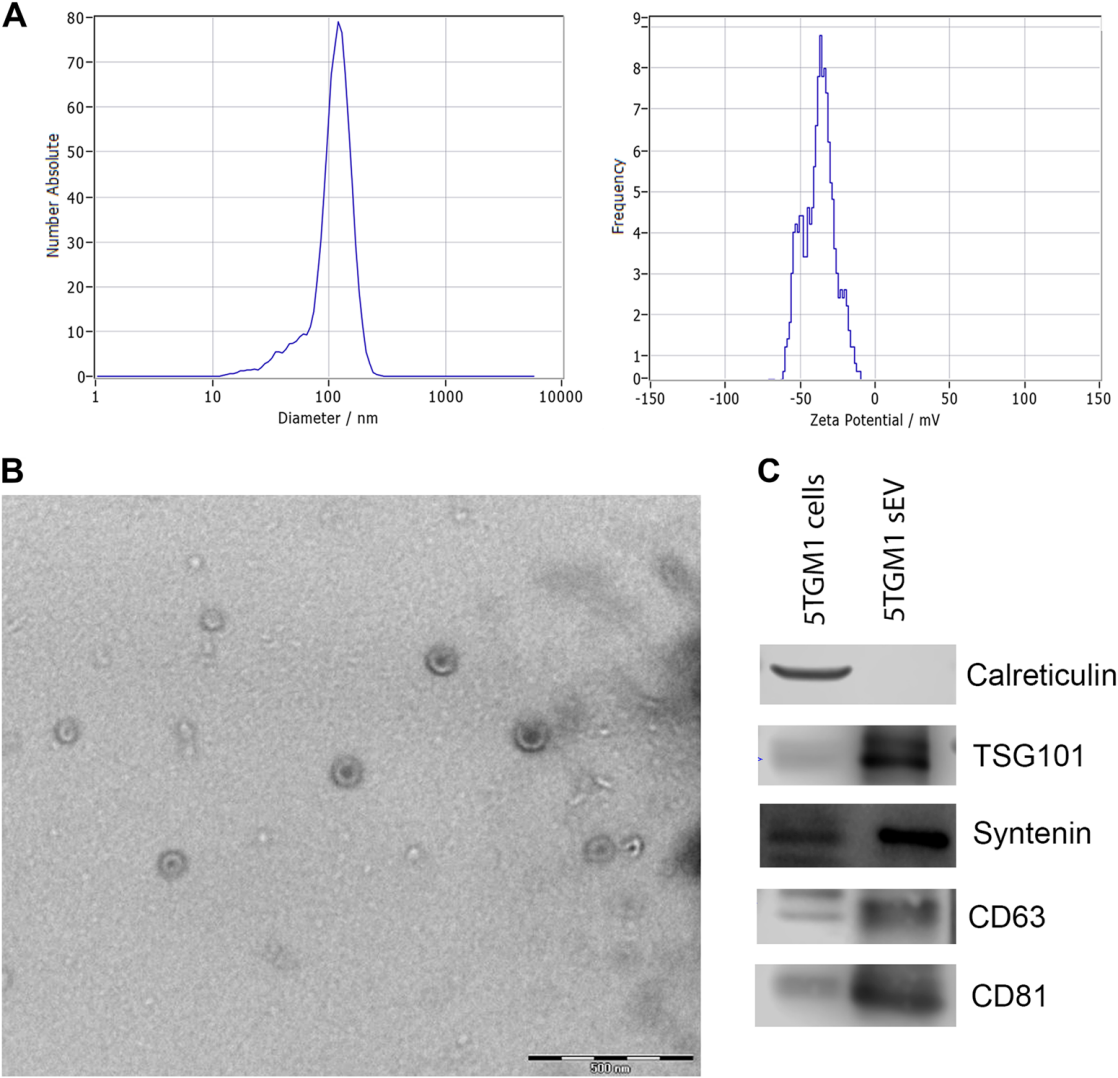
Characterization of extracellular vesicles. **a** Nanoparticle tracking analysis by Zetaview of isolated EVs show a mean diameter of 114.4 nm (SD 36.9 nm) with a maximum diameter of 153 nm; analysis of zeta potential show a negative potential of these vesicles. The Zetaview was calibrated using standard beads of 100 nm. EVs were dissolved in PBS and PBS was used as a negative control. 11 frames per sample were analyzed. **b** Transmission electron microscopy analysis of sEVs isolated by ExoQuick protocol show the presence of vesicles with the appropriate size and morphology. **c** Western Blot analysis of extracellular vesicles show the absence of calreticulin (intracellular protein, absent in EVs/exosomes) and presence of tetraspanins CD81 and CD63 as well as endosomal markers TSG101 and Syntenin, enriched in exosomes. One experiment representative of 3 is shown

We first isolated small EVs with a size of around 114 nm, as determined by nanoparticle tracking analysis, from the conditioned medium of 5T33MMvt and 5TGM1 cells (Fig. 1a). Figure 1b demonstrates the double membrane of the vesicles, visualized by transmission electron microscopy. By western blot, we detected the presence of the endosomal proteins TSG101 and Syntenin, and the tetraspanins CD63 and CD81 (Fig. 1c). The presence of these proteins together with their size, indicate that the EVs are from endosomal origin, and can be called exosomes.^15^

### 5TGM1 sEVs induce osteolysis in vivo

Malignant cells use EVs as a mechanism of cell–cell communication, inducing a premetastatic niche in solid tumors such as melanoma^16^. In different hematological cancers including chronic myelogenous leukemia, acute myeloid leukemia and MM, EVs can create a permissive BM microenvironment with enhanced angiogenesis^17,18^, increased immune suppression^8^ and increased osteolysis^19^.

**Fig. 2:**
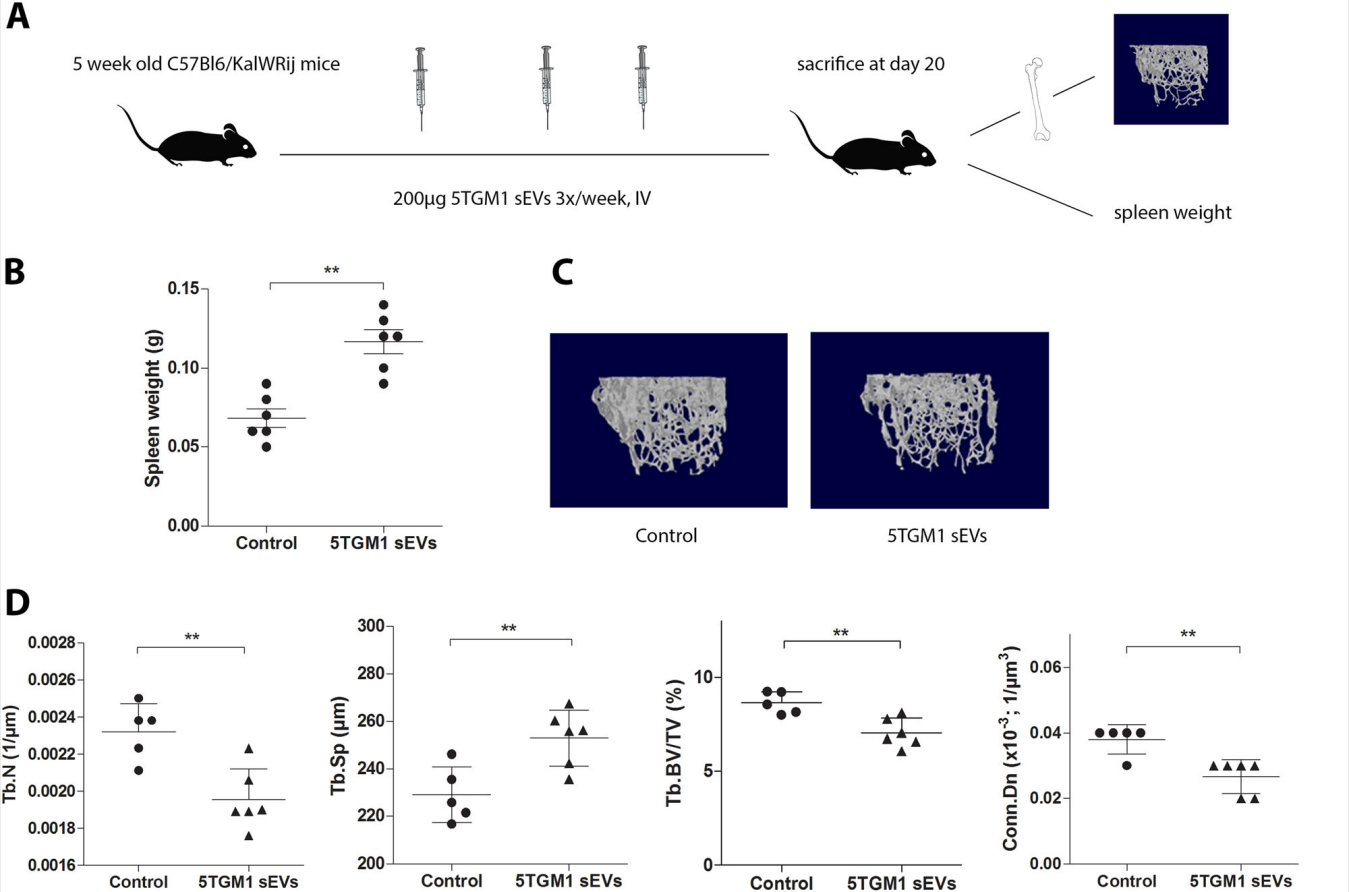
5TGM1 sEVs induce osteolysis in vivo in C57Bl6/KalWRij mice. **a** 5-week old C57Bl6/KalWRij mice (*n* = 6) were injected intravenously three times per week with 200 µg of exosomes, for three weeks. At day 20, all mice were sacrificed and femurs and spleen were isolated. **b** Spleens of mice injected with 5TGM1 sEVs nearly doubled in size compared to controls injected with PBS. **c** Representative 3D-reconstruction of trabecular bone of femurs of control mice vs. 5TGM1 sEVs injected mice. **d** Morphometric µCT analysis shows a significant lower trabecular number (Tb. N) and higher Trabecular separation (Tb Sp.), leading to a significantly lower trabecular bone volume and a lower connectivity density in mice injected with 5TGM1 sEVs. Experiment was repeated twice, once as a pilot study with *n* = 3. Final experiment is shown (*n* = 6). ** = *p* < 0.01

Here we first wished to confirm the effects of MM sEVs in vivo on osteolysis by injecting 5-week old mice with 5TGM1 sEVs intravenously for three weeks (Fig. 2a). We chose 5TGM1 sEVs since this model has been shown to develop osteolytic lesions^20^.

Mice injected with MM sEVs showed an enlarged spleen, similar to mice inoculated with MM cells (Fig. 2b). To examine osteolysis, we analyzed the femurs for bone density and volume by micro-computed tomography (µCT) (Fig. 2c, d). Quantitative µCT analysis (Fig. 2d) showed that 5TGM1 sEVs could induce osteolysis in a similar destructive pattern as the 5TGM1 cells themselves, albeit at a lesser extent (19% decrease in trabecular bone volume for sEVs versus 40% for 5TGM1 cells, supplementary Fig. 1A). Specifically, we saw a significant reduction of trabecular bone volume (Tb.BV/TV) due to a decrease in number of trabeculae (Tb. N) and an increase in trabecular separation (Tb.Sp). As a result, trabecular connectivity density (Conn. Dn) significantly decreased in mice injected with 5TGM1 sEVs.

### 5TGM1 sEVs induce differentiation of RAW 264.7 cells into osteoclasts and increase their bone resorptive activity

**Fig. 3:**
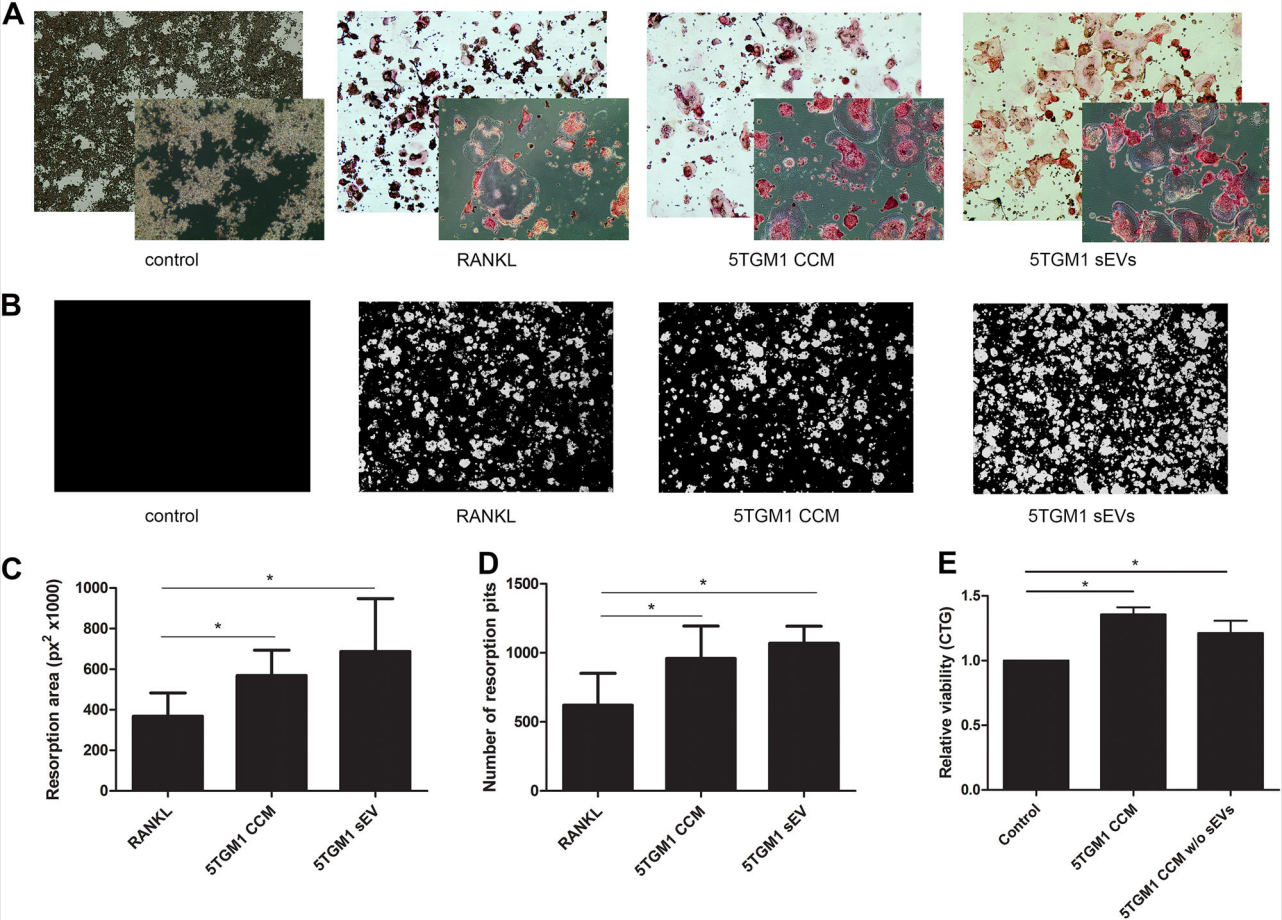
5TGM1 sEVs aid in differentiation of RAW264.7 cells to osteoclasts, and increase their resorptive activity. **a** Representative images of TRAP-stained RAW264.7 osteoclast cultures incubated with RANKL (30 ng/ml) on day 1 in all conditions. At day 4, medium was refreshed and either 5TGM1 CCM, or 5TGM1 sEVs (100 µg/ml) was added. ×4 and ×10 magnifications are shown. **b** Representative images of resorption pits (Von Kossa staining) generated by RAW264.7 osteoclasts incubated with either 5TGM1 CCM, 5TGM1 sEVs (100 µg/ml) or RANKL (positive control) (×40 magnification). **c** Quantification of the matrix resorption area in RAW264.7-derived osteoclast cultures. **d** Quantification of the number of resorption pits per field of view (N. pits/FOV). **e** RAW264.7 osteoclast cultures were cultured with 5TGM1 concentrated conditioned medium (CCM) or medium deprived of sEVs. Relative viability after 24 h was determined by a Cell Titer Glo® luminescence assay. Control = serum-free medium. Mean value + SD are shown for 3 independent experiments, *: *p* < 0.05

Since previous reports indicated a supportive effect of MM sEVs on osteoclast differentiation and activity^11,12^, we wished to assess the effect of 5TGM1 sEVs on the differentiation and activation of RAW264.7 cells. 5TGM1 sEVs did not induce differentiation of RAW264.7 cells toward osteoclasts (results not shown), however when these EVs were added to already differentiated osteoclasts (after prior stimulation with RANK-ligand) there was an increased resorptive activity compared to RANK-ligand alone and osteoclasts appeared larger (Fig. 3a). The increased resorption resulted from an increase in the absolute number and size of resorption pits (Fig. 3b). Compared to RANK-L, the number of resorption pits significantly increased (1068 vs 621) while pits were larger (686 vs 367 px^2^ *1000). Interestingly, results obtained by adding 5TGM1 CCM after prior RANK-L stimulation, tended to be lower compared to the results obtained with 5TGM1-sEVs (Fig. 3c, d). To evaluate whether the effects of 5TGM1 CCM were mainly the result of 5TGM1 sEVs, we evaluated the effect of 5TGM1 CCM of which we removed sEVs through ultracentrifugation, on the viability of differentiated RAW264.7. Figure 3e demonstrates that 5TGM1 CCM stripped of sEVs was still able to induce increased viability, indicating that in CCM there are osteoclasts stimulating cytokines present, apart from sEVs.

### 5TGM1 sEVs induce apoptosis and inhibit differentiation of osteoblasts in vitro

To further analyze the observed osteolytic in vivo activity of 5TGM1 sEVs, we focused on possible effects on osteoblasts in vitro by performing viability, apoptosis and differentiation assays on MC3T3-E1 cells. These cells are a pre-osteoblast murine cell line, and can be differentiated to osteoblasts by the addition of β-glycerophosphate and ascorbic acid^21^.

**Fig. 4:**
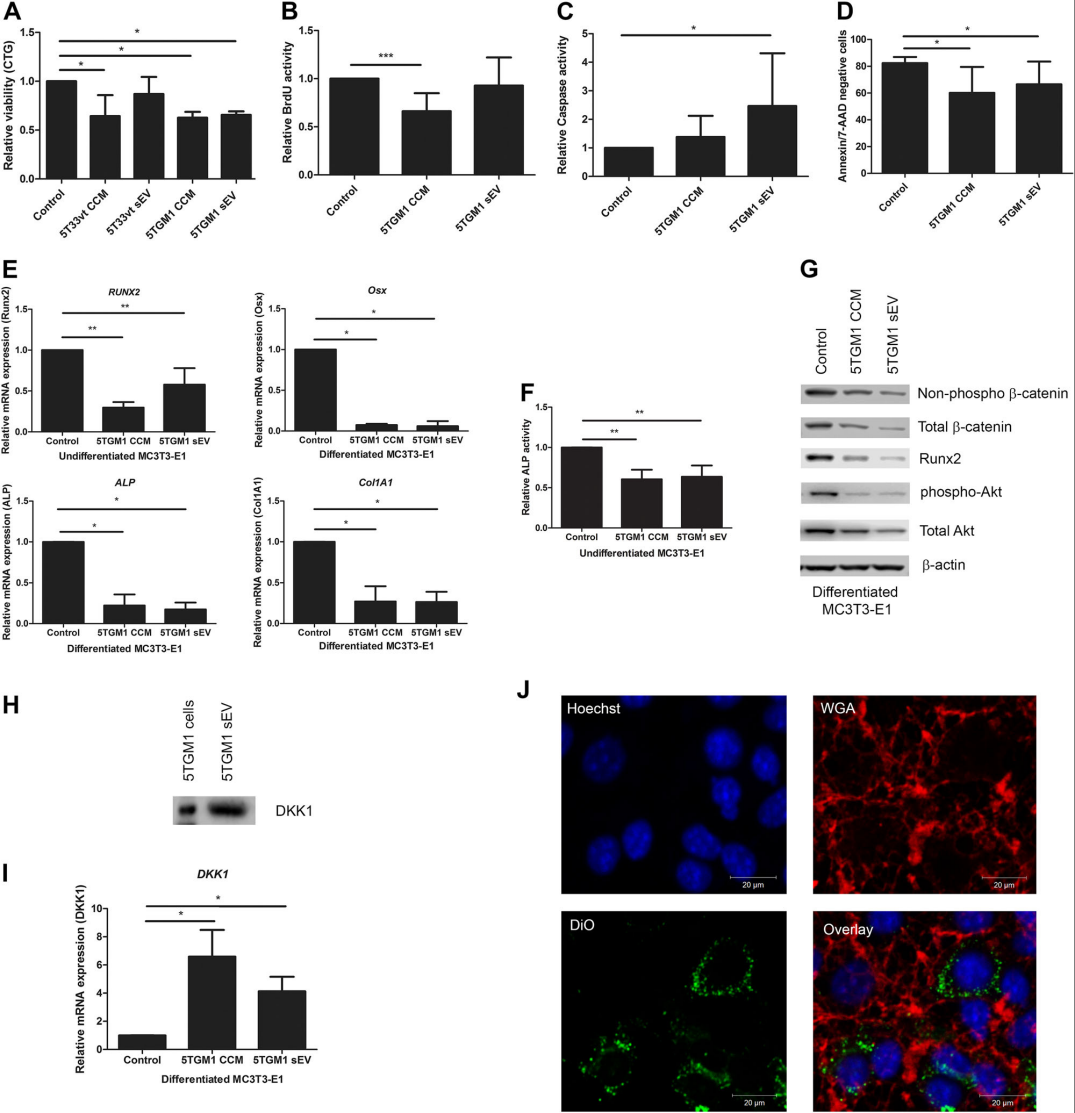
5TGM1 sEVs induce apoptosis and inhibit differentiation of MC3T3-E1 cells. **a**–**d**. MC3T3-E1 cells were cultured with 5TGM1/5T33vt concentrated conditioned medium (CCM) or 5TGM1/5T33vt sEVs (100 µg/ml) in serum-free medium. Control = serum-free medium. **a**. Relative viability after 48 h was determined by a Cell Titer Glo® luminescence assay. **b**. Relative proliferation was determined after 24 h with a BrdU incorporation assay. **c**. Relative caspase activity determined by CaspaseGlo after 24 h. **d**. Living MC3T3-E1 cells after culture for 24 h determined by Flow Cytometry analysis (AnnV/7AAD negative cells). **e** Relative mRNA expression was determined by qRT-PCR for RUNX2 in undifferentiated MC3T3-E1 cells. Osterix (Osx), Collagen 1A1 (Col1A1) and Alkaline phosphatase (ALP) expression were compared in differentiated MC3T3-E1 cells after 24 h of culture with the three previously described conditions. **f** Relative ALP activity was measured after 24 h by an ALP yellow liquid substrate assay. ALP activity was normalized to total amount of protein. **g** Western blot analysis from MC3T3-E1 cell lysates after 24 h of culture with either serum-free medium, 5TGM1 CCM or 5TGM1 sEVs, representing downregulation of Wnt signaling pathway, Runx2 expression, and loss of phospho-Akt. **h** Western blot analysis shows the presence of DKK1 on 5TGM1 sEVs and in the 5TGM1 cell lysate (5TGM1 cells). **i** qRT-PCR shows upregulation of DKK1 in differentiated osteoblasts cultured with 5TGM1 CCM or sEVs for 24 h. Control = serum-free medium. **j** Visualization of uptake of 5TGM1 sEVs by MC3T3-E1 cells after 24 h by confocal microscopy. Nuclei and membranes of MC3T3-E1 cells were stained by Hoechst and WGA respectively. 5TGM1 sEVs were labeled with DiO-membrane staining. Bars represent mean ± SD. Experiments were repeated at least three times independently. * = *p* < 0.05

We first tested the effects of 5TGM1 sEVs on the viability of the MC3T3-E1 cells. We compared the effect of CCM and sEVs from 5TGM1 cells. Both were able to significantly reduce the viability of MC3T3-E1 cells at 48 h (Fig. 4a). To determine whether this was the result of an effect on apoptosis or proliferation, we performed BrdU incorporation assays (Fig. 4b) and measured apoptosis by Caspase-Glo® 3/7 Assay (Fig. 4c) and Annexin V/7-AAD staining (Fig. 4d). Interestingly, while CCM had a more profound effect on proliferation (34% vs 8%, Fig. 4b), 5TGM1 sEVs induced more apoptosis of the MC3T3-E1 cells (1.4 fold vs 2.5 fold increase as measured by Caspase-Glo® 3/7 Assay), indicating that other factors in CCM govern this anti-proliferative effect. By western blot analysis, we also saw a lower expression of pAkt/total Akt on protein level confirming reduced survival (Fig. 4g).

To further confirm the specific activity of 5TGM1 sEVs on osteoblasts, we compared their effect with that of 5T33MMvt sEVs, which are known to have less osteolytic capacities when injected in vivo. We could confirm this finding in vitro where we found that 5T33MMvt sEVs had no significant effect on osteoblast viability, in contrast to the 5T33MMvt CCM (Fig. 4a).

In a next step, we evaluated the role of MM sEVs in osteoblast functionality by first investigating the differentiation capacity of osteoblasts. To analyze effects on early differentiation markers (Runx 2) we used undifferentiated MC3T3-E1 cells; by contrast for late differentiation markers (Osterix, Collagen 1 A1) we first differentiated the MC3T3-E1 cells for two weeks; followed by culture with sEVs or CCM. Already after 24 h, the expression of Runx2, the master regulator gene for osteoblast differentiation was decreased; as shown by RT-PCR analysis as well as WB analysis (Fig. 4e, g). Similarly, we saw a decreased Osterix and Collagen 1 A1 gene expression when adding sEVs to the culture medium (Fig. 4e). Also, alkaline phosphatase (ALP) expression and activity of the osteoblasts was diminished by sEVs and CCM, as measured by RT-PCR and the colorimetric ALP assay (Fig. 4e, f). Since ALP and Collagen 1 A1 are proteins produced by terminally differentiated osteoblasts, necessary for the production of extracellular matrix, their downregulation illustrates that the osteoblasts also lost in vitro at least part of their functional capacities, due to the exposure to 5TGM1 sEVs.

### Inhibition of osteoblast differentiation by 5TGM1 sEVs occurs through downregulation of the Wnt pathway

The downregulation of the Wnt pathway has been described to be involved in the inhibition of osteoblast differentiation by MM cells and is triggered by binding of either DKK1 or the soluble Frizzle Related protein to the osteoblast cells. This induces a phosphorylation of β-catenin, making it inactive. Only active β-catenin will transfer to the nucleus and induce transcription of osteoblast differentiation genes^22^.

We hypothesized that 5TGM1 sEVs might also execute their effects by inhibition of the Wnt pathway. Indeed, WB analysis confirmed the downregulation of total and active β-catenin in MC3T3 cells cultured with 5TGM1 sEVs (Fig. 4g). We then analyzed the expression of DKK1 and found that not only do 5TGM1 sEVs contain DKK1; they also induced upregulation of DKK-1 expression in osteoblasts (Fig. 4h, i). We next examined the mechanisms behind this inhibition. We visualized the interaction between DiD-labeled 5TGM1 sEVs and osteoblasts by confocal imaging, and could see an uptake of the fluorescent-labeled sEVs after 24 h, confirming the interaction of the sEVs with the osteoblasts (Fig. 4j).

### GW4869 inhibits the secretion of exosomes by 5TGM1 in vitro

**Fig. 5:**
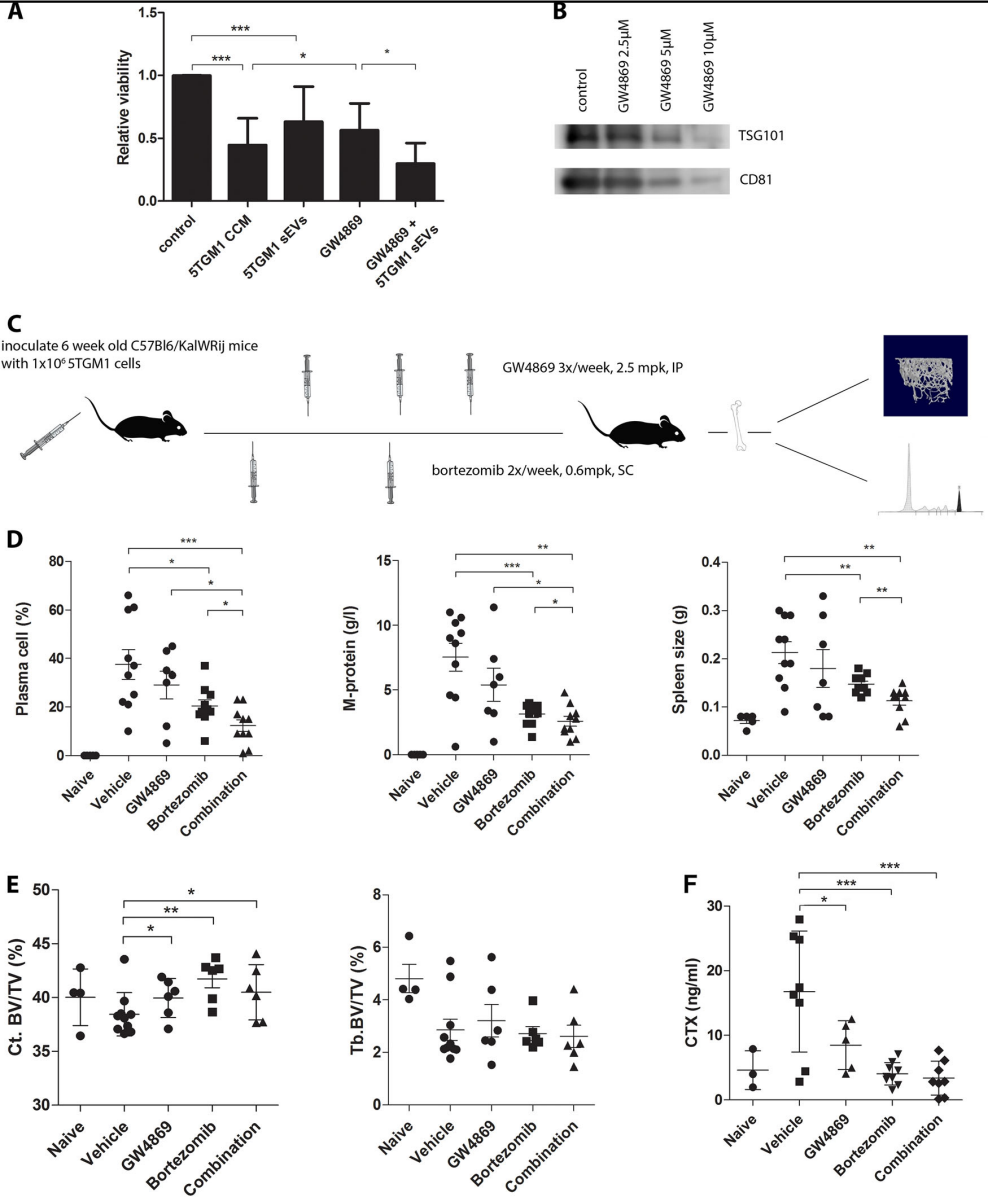
Inhibiting the secretion of exosomes in 5TGM1 mice with GW4869 reduces tumor load when combined with standard-of-care treatment bortezomib. **a** Relative viability of MC3T3-E1 was measured by a Cell Titer Glo assay. Cells were cultured in 5TGM1 concentrated conditioned medium (CCM), 5TGM1 sEVs (100 µg/ml); 5TGM1 CCM collected after treatment of 5TGM1 cells with GW4869 (10 µM) for 24 h (GW4869), reducing exosome secretion and finally a combination of 5TGM1 GW4869 CCM and 5TGM1 sEVs (100 µg/ml) (GW4869 + 5TGM1 sEVs). Bars represent mean ± SD. Experiments were repeated at least three times. * = *p* < 0.05, *** = *p* < 0.001. **b** Western blot analysis of 5TGM1 CCM after treatment for 24 h with an indicated dosage of GW4869, showing a reduction in exosomal markers TSG101 and CD81. **c** 6-week old mice were inoculated with 1 million 5TGM1 cells on day 1. Treatment with bortezomib (0.6 mpk, two times per week, SC) and/or GW4869 (2.5 mpk, three times per week, IP) started the next day. *N* = 10 for each treatment group. At day 25 all mice were sacrificed. **d** Spleen, serum and bone marrow (BM) were collected for analysis of M-protein (protein electrophoresis) and BM plasmacytosis. **e** Quantitative µCT analysis was performed for naive and vehicle mice. For mice from the treatment groups, six mice with representative tumor load for the rest of the group were selected for µCT analysis. Shown are cortical bone volume (Ct. BV/TV) and trabecular bone volume (Tb. BV/TV). * = *p* < 0.05, ** = *p* < 0.01. **f** Levels of serum circulating type I collagen degradation product (Collagen C-terminal telopeptide or CTX) was measured by the CTX ELISA kit (cf. supplementary materials) in all treatment groups. Serum was diluted 1:10. * = *p* < 0.05, *** = *p* < 0.001

We finally wished to examine the effects of blocking exosome secretion on osteoblast viability in vitro and the BM microenvironment in vivo. Therefore, we tested the neutral sphingomyelinase inhibitor GW4869 for its inhibitory effect on exosome secretion. This compound partly inhibits the secretion of exosomes by the ceramide pathway^23^. In vitro, we could clearly see at 10 µM of GW4869 a downregulation of exosomal markers by WB, indicating a lower production of exosomes by 5TGM1 cells treated with the inhibitor (Fig. 5a). Furthermore, we saw an increased osteoblast cell viability when using CM from 5TGM1 cells treated with GW4869 and thus containing less exosomes. Cell viability was again decreased when adding 5TGM1 sEVs to the GW4869-treated CM. This indicates that the effects on cell viability seen by the addition of 5TGM1 CCM is at least partly due to the presence of exosomes, and that these exosomes can be eliminated from the CCM by treating the cells with GW4869 (Fig. 5b).

### Inhibiting the secretion of exosomes in 5TGM1 mice reduces tumor load when combined with standard-of-care treatment bortezomib

Myeloma exosomes, as well as stromal cell exosomes, create a permissive microenvironment in the BM, resulting in drug resistance to standard-of-care agents, including bortezomib^7,8,11^.

To evaluate the effects on tumor load and the altered microenvironment in vivo, we wanted to inhibit exosome secretion in myeloma-bearing mice, hypothesizing that in combination with bortezomib, this would have a significant impact on tumor load. Moreover, we wanted to look at angiogenesis and osteolysis in the microenvironment when inhibiting all exosome secretion (from both stromal and myeloma cells) in myeloma-bearing mice.

We first analyzed whether GW4869 had any direct effects on 5TGM1 cells and osteoclasts in vitro. We found no detrimental effects on cell viability on either cell type at the used dose of 10 µM (supplementary Fig. 1B), indicating that the effect of GW4869 is solely through blocking of exosome secretion.

We inoculated 6-week old KaLwRrij mice with 1 million 5TGM1 cells, and treated mice with GW4869, bortezomib or a combination (Fig. 5c). Results on tumor load are illustrated by plasma cell count in the bone marrow, M-protein spike analyzed by protein electrophoresis and spleen size (Fig. 5d). As expected, GW4869 did not exhibit clear effects on tumor load, illustrating that GW4869 itself is not toxic to the myeloma cells. We could, however, see a significantly better effect on tumor load with the combination of GW4869 and bortezomib than with either of the two single agents, indicating that inhibiting exosome secretion in the microenvironment strengthens the anti-tumor effects of bortezomib.

Furthermore, the quantitative µCT analysis revealed osteolytic changes in 5TGM1 bearing mice with a decreased trabecular and cortical bone volume. Inhibiting exosome secretion in these animals using GW4869 showed a significant protective effect on cortical bone volume, even though tumor load was not significantly affected. These data were supported by a decreased level of circulating type 1 collagen degradation product, a serum marker for osteoclast activity. This indicates that inhibiting exosome secretion in the MM microenvironment has a protective effect on MMBD, not associated with effects on tumor load (Fig. 5e, f).

Finally, we have previously demonstrated that myeloma cells induce angiogenesis in the BM, partly by exosome secretion^8^. Myeloma tumor load normally correlates well with angiogenesis, quantified by measuring CD31 staining of the endothelial cells lining the neovasculature in the BM (microvessel density or MVD). Inhibiting exosome secretion by GW4869, combined with bortezomib treatment resulted in a significant decrease of MVD. (supplementary Fig. 1C).

## Discussion

MM bone disease is an important clinical issue in virtually all MM patients, responsible for significant morbidity and decline in their quality of life. Osteolytic lesions, bone pain, and pathological fractures are the result of an imbalance in bone remodeling, favoring bone resorption over bone formation. On the other hand, exosomes, and by extension other EVs, enable MM disease progression by inducing angiogenesis, immunosuppression, and drug resistance. Moreover, here we demonstrate for the first time in a clinically relevant model the involvement of exosomes in osteolysis. Exosome secretion might therefore be an attractive target to simultaneously target MM development and bone disease.

Exosomes are small EVs, formed inside multivesicular bodies (MVB) and are actively secreted by the cell through fusion of the MVB with the cell membrane. Other EVs, such as microvesicles, are formed by a direct budding from the plasma membrane. They can range in size from 30–1000 nm. Since none of the presently available EV isolation methods can differentiate between real exosomes, derived from the endosomal pathway, or other small EVs, there is a consensus to simply name them ‘small EVs’ instead of exosomes. In this study, we used an ExoQuick isolation method, with some additional centrifugation and filter steps to increase exosome purity. However, we repeated the in vitro experiments on osteoblasts with sEVs isolated by OptiPrep Density ultracentrifugation, to ensure that the effects observed were not the result of secreted proteins co-isolated with the ExoQuick method (supplementary Fig. 1D). We next demonstrated the capacity of MM exosomes to induce osteolysis in vivo, in a similar destructive pattern as when mice were inoculated with 5TGM1 cells. This might indicate that MM exosomes can specifically home and create a niche in the BM, as we have previously described using MM-BMSC exosomes^7^, and was also recently demonstrated with AML exosomes^19^. Another recent study determined the osteolytic effects of sEVs from the human JJN3 line when injected directly into the calvaria of NOD-SCID mice^11^. While this study confirms the effect of human MM sEVs on osteolysis, we believe that our syngeneic model is closer to the clinical situation. Moreover, intravenous injection of sEVs is more appropriate since tumoral sEVs can be distributed by blood to distant sites and execute their effects without the presence of MM cells, hereby prepping the BM for future invasion. Furthermore, Zahoor et al. found that the presence of IL-32 on the sEVs was imperative for the observed effect. The 5TGM1 model, however, does not express IL-32, based on our microarray data (unpublished data), indicating that other factors also play a role.

It is well known that osteoclasts are stimulated by MM cells through various molecules, including IL-3, IL-6, RANKL, M-CSF, and TNF-α. Vice versa, osteoclasts support the survival and progression of the MM cells. Previous studies have hinted at the implication of EVs in these processes. Here, we demonstrated that while 5TGM1 sEVs had limited effect on osteoclast differentiation, they greatly increased their resorptive activity.

We then evaluated the effects of 5TGM1 sEVs on the pre-osteoblast MC3T3-E1 line, and observed caspase-mediated apoptosis and a decrease in the expression of various genes related to osteoblast differentiation. The effects of 5TGM1 sEVs on viability of MC3T3 cells were more pronounced than those of 5T33vt sEVs. This is in accordance with the osteolytic potential of the cells of origin. When comparing the effects of 5TGM1 sEVs to the effects of 5TGM1 CCM we found more pronounced effects with CCM which is not surprising since this also contains other factors including interleukins, matrix metalloproteinases, RANKL, and M-CSF. When blocking sEVs secretion with GW4869 the effect of the CCM was reduced, confirming the involvement of sEVs. While there is extensive research about the inhibition of osteoblastogenesis and osteoblastic activity by MM cells through secreted factors^24^, we are the first ones to indicate the role sEVs play in this process.

We furthermore found that 5TGM1 sEVs caused a downregulation of Runx2 in undifferentiated MC3T3-E1 cells. Runx2 is the master regulator of the early osteoblast differentiation process. In differentiated MC3T3-E1 cells, we observed a lower expression of Osterix, Collagen 1A1 and ALP. These effects seem to be the result of an inactivation of the Wnt signaling pathway, as shown by downregulation of total and active β-catenin. The Wnt pathway is responsible in osteoblasts for their proliferation, differentiation, and activity^25^. Myeloma cells are known to produce Wnt signaling inhibitors such as soluble Frizzled-related protein 2 and 3, and Dickkopf-1 (DKK-1), hereby impeding the normal osteogenesis. We could demonstrate the presence of DKK-1 in the isolated sEV fraction, and saw that DKK-1 was upregulated in MC3T3-E1 cells cultured for 24 h with 5TGM1 sEVs, indicating the possible transfer of transcription factors. DKK-1 inhibitors have been used in the past to stimulate bone mass in MM, with good preclinical results in the 5TMM model^26^.

Finally, the ultimate goal of this study was to inhibit exosome secretion in vivo and analyze the effects on tumor load, and on the microenvironment. Exosomes from both stromal and myeloma cells play various important roles in the MM microenvironment. Importantly, stromal exosomes induce drug resistance to bortezomib, one of the most important first-line standard-of-care agents in MM^7^. MM exosomes, on the other hand, induce angiogenesis, immunosuppression, and osteolysis^6,8^. Therefore, it seemed interesting to inhibit all exosome secretion in the microenvironment. We used GW4869, which is a neutral sphingomyelinase inhibitor that inhibits the formation of exosomes by the ceramide pathway. We first demonstrated the efficacy of the inhibitor in vitro. Although previous reports claimed a toxicity of this agent in certain MM cell lines^27^, we found no negative effects at the dosage used nor direct effects on osteoclasts.

GW4869 has already been used in vivo to inhibit exosome secretion in different disease models^28–31^. Here, when used as a single agent, GW4869 only had a modest effect on tumor load. However, combination treatment with bortezomib led to a significant effect on tumor load, indicating that exosome secretion in the BM microenvironment mainly induces drug resistance and therefore impedes the effects by bortezomib as we have previously demonstrated in vitro^7^.

Although GW4869 in itself did not significantly lower tumor load, we did see an effect on angiogenesis and osteolysis. This confirms the involvement of MM exosomes in angiogenesis as we have previously determined^8^. Effects on osteolysis were mainly restricted to a higher cortical bone volume in mice treated with either GW4869, bortezomib, or the combination of both. It is unusual to see an effect on cortical bone volume without seeing an effect on trabecular bone volume in myeloma, since the remodeling time of cortical bone is slower than trabecular bone. However, clinically spoken it might be more relevant to see an effect on cortical bone volume, since a stronger cortex will prevent fractures. Furthermore, we did see a clear effect on a circulating collagen type I, which, as a serum marker, gives a more general overview of osteoclast activity and osteolysis rather than µCT analysis, which can overlook focal osteolytic lesions.

In conclusion, we can state that we established the role of 5TGM1 sEVs in MM bone disease in a relevant syngeneic model with high translational value when compared to other models which use human cells/exosomes implanted in a murine microenvironment. We demonstrated that this is both the result of the activation of osteoclasts and inhibition of osteoblasts, partly through the transfer of DKK-1. Blocking exosome secretion in vivo not only led to a reduction in osteolysis but also a significant anti-tumor effect when combined with bortezomib, indicating that exosome secretion is a possible novel therapeutic target in MM.

## Electronic supplementary material

supplemental information without highlights

supplemental figure 1
