# Supplementary material for: Exosomes play a role in multiple myeloma bone disease and tumor development by targeting osteoclasts and osteoblasts
Source: Blood Cancer J. 2018 Nov 8;8(11):105. doi: 10.1038/s41408-018-0139-7 (PMC6224554; doi:10.1038/s41408-018-0139-7)
Supplement: Supplementary file 1 — supplemental information without highlights [file 41408_2018_139_MOESM1_ESM.docx]

Supplementary Information

Supplemental material and methods

Cell lines

The murine MM cell line 5TGM1 (kindly provided by dr. Kay Oyajobi, Vanderbilt University, Nashville, TN, USA) was cultured in RPMI-1640 (Lonza, Basel, Switzerland) supplemented with 10% fetal bovine serum (FBS) (Hycone, Logan, UT, USA), 2mM L-glutamine, 1mM sodium pyruvate and 1% penicillin/streptomycin (Thermo Fisher Scientific, Waltham, MA, USA) at 37°C in 5% CO_2_.

For collection of concentrated conditioned medium, 5TGM1 cells were cultured during 24h in serum-free medium at a density of 2 million / ml. Medium was concentrated 10 times using a 150 kD concentrator (Thermo Scientific, Waltham, MA, USA) and sterilized using a 0.22 µM pore filter (Carl Roth, Karlsruhe, Germany).

The pre-osteoblast murine cell line MC3T3-E1 was cultured in αMEM medium (Thermo Fisher Scientific), supplemented with 10% FBS, 2mM L-glutamine and 1% penicillin/streptomycin (P/S).

EV isolation by Optiprep density gradient ultracentrifugation.

A discontinuous iodixanol gradient was prepared by overlaying solutions of 5, 10, 20 and 40% iodixanol, as previously described.(1)

The gradient was formed by layering 3 ml of 40%, 3 ml of 20%, 3 ml of 10% and 2.5 ml of 5% solutions on top of each other in a 13.2 ml open top polyallomer tube (Ref 331372, Beckman Coulter, Brea, CA, USA). 500 µl CCM sample was overlaid onto the top of the gradient which was then centrifuged for 18 hours at 100 000 g and 4°C (SW 41 rotor, Beckman Coulter). Gradient fractions of 1ml were collected from the top of the gradient, and fraction 6 and 7 were diluted to 13 ml in PBS and centrifuged for 3 hours at 100 000 g and 4°C. Pellets were resuspended in 50 µl PBS.

Transmission electron microscopy

Small EVs were fixed in 2% paraformaldehyde and absorbed to a formvar-carbon coated grid. After adsorption, the grids were transferred to 1% glutaraldehyde, washed 8 times with Milli-Q water and negatively stained with 2% uranyl acetate. The grids were visualized on a TECNAI 10 transmission electron microscope (Philips, Amsterdam, The Netherlands) at 80kV and the images were captured using iTEM software (Olympus Soft Imaging Solutions, Münster, Germany).

Micro-computed tomography

µCT was performed on distal femurs of mice with the Skyscan 1172 system (Bruker, Kontich, Belgium as described previously. (2) 3D models of bones were generated using CTVol software (Bruker).

Western Blotting

MC3T3-E1 were seeded in 6-well plates and differentiated for 14 days using osteogenic medium. After 14 days, medium was replaced with control medium, 5TGM1 conditioned medium or 5TGM1 sEVs during 48 hours after which cells were lysed in lysis buffer containing 50mM Tris, 150mM NaCl, 1% Nonidet P40, and 0.25% sodium deoxycholate. The following protease and phosphatase inhibitors were added: 4mM Na3VO4, 1mM Na4P2O7, 2 µg/mL aprotinin, 50 µg/mL leupeptin, 500 µg/mL trypsin inhibitor, 10µMbenzamidine, 2.5mM pnp benzoate (all from Sigma-Aldrich), 50mM NaF,5mMethylenediaminetetraacetic acid (both from VWR International), 1mM 4-(2-aminoethyl) benzenesulfonyl fluoride hydrochloride, and 50µg/mL pepstatin A (both from ICN).

Western Blot analysis on these cell lysates was performed as previously described (3). Chemiluminescence was visualized and analyzed using Li-Cor Odyssey Fc (Westburg, Leusden, Netherlands).

Antibodies used for osteoblast analysis were: RUNX2 (D1L7F, #12556), SMAD5 (#9517), β-CATENIN (D10A8, #8480) and NON-PHOSPHO-βCATENIN (Ser33/37/Thr41, D13A1, #8814), and β-ACTIN (#4967, all from Cell Signaling Technology, and DKK-1 (PA5-23187) from Thermo Fisher Scientific. For exosome characterization we used TSG101 (C-2, sc7964), CD63 (H-193, sc-15363), CD81 (B-11, sc-166029) from Santa Cruz Biotechnology, SYNTENIN (ab 19903, Abcam) and CALRETICULIN (#2891, Cell Signaling Technology).

Viability, apoptosis and proliferation assay

MC3T3-E1 or differentiated RAW264.7 cells were seeded in a 96-well plate and cultured in either no serum medium, 5TGM1 concentrated conditioned no serum medium (CCM), 5TGM1 CCM depleted of sEVs, or no serum medium containing 5TGM1 sEVs (100µg/ml).

Viability was measured after 48h by a CellTiter Glo® Luminescent Cell Viability Assay (Promega, Madison, WI, USA). Apoptosis was measured after 24h by a Caspase-Glo 3/7 Assay (Promega). Luminescence was measured using a Glomax luminometer (Promega). Viable cells were quantified after 24h by a AnnexinV-FITC and 7-AAD staining (Becton Dickinson) by flow cytometry analysis on a FACSCanto flow cytometer (BD Biosciences, Belgium)

MC3T3-E1 proliferation was determined by using the BrdU Cell Proliferation Assay Kit (#6813, Cell Signaling Technology). BrdU was added after 24h of adding 5TGM1 conditioned medium or 5TGM1 sEVs. The assay was started 24h after adding BrdU, according to manufacturer’s instructions. Absorbance was read at 450 nm using the BioRad iMark™ Microplate Absorbance reader (BioRad laboratories, Temse, Belgium).

Quantitative real-time PCR

RNA was isolated using the RNeasy kit (Qiagen, Antwerp, Belgium) and converted to cDNA by the Verso cDNA Synthesis Kit (ThermoFisher Scientific, Waltham, MA, USA). Gene-specific primer sequences were as follows, Runx2: forward (5’ – ACT CTT CTG GAG CCG TTT ATG – 3’), reverse ( 5’ – GTG AAT CTG GCC ATG TTT GTG – 3’), mouse Osterix: forward (5’ – TGC GCC AGG AGT AAA GAA TAG – 3’), reverse (5’ – CCT GAC CCG TCA TCA TAA CTT AG 3’), mouse ALP: forward (5’ – GGA ATA CGA ACR GGA TGA GAA GG -3’), reverse (5’- GGT TCC AGA CAT AGT GGG AAT G – 3’-), mouse Collagen 1A1: forward (5’ – TTC TAG TTC CTG GGC CTA TCT - 3’), reverse (5’ – GAT GCA GGA CAG ACC AAG AG – 3’); mouse DKK1: forward (5’ – CCA TTC TGG CCA ACT CTT TCT A – 3’), reverse (5’ – GCA TTC CCT CCC TTC CAA TAA – 3’); all from Integrated DNA Technologies. Expression level of mRNA was quantified by qRT-PCR with PowerUp™ SYBR® Green Master Mix (Thermo Fisher Scientific) using the ABI 7900TH Real-Time PCR System (Applied Biosystems). ABL was included as an internal control. Relative mRNA expression normalized to ABL was carried out using the 2-ΔΔCt method.

CD31+ staining and microvessel density

The contralateral femur of 5TGM1 mice was  incubated in zinc fixative (0.1 m Tris, 3 mM calcium acetate, 0.27 m zinc acetate, and 0.037 m zinc chloride), decalcified in FE10 (0.27 m EDTA, 0.3 m NaOH, 2% formalin), embedded in paraffin and 5 μm sections were cut. Sections were stained for the presence of CD31 (PECAM-1) to identify the presence of microvessels. For CD31 retrieval, sections were incubated in trypsinand blocked with normal goat serum for 30 min. Sections were then incubated with a rat anti-CD31 antibody (PECAM-1; BD Biosciences), or an appropriate isotype control, at 4°C overnight. The sections were washed and incubated with a goat anti-rat antibody conjugated with biotin (1/100 dilution; BD Biosciences). The presence of bound antibody was detected with a streptavidin–HRP conjugate in combination with tyramide signal amplification (NEN Life Science Products, Boston, MA, USA). Diaminobenzidine was used as substrate. The number of blood vessels and sinusoids (per 0.22 mm^2^) were counted in a tumour-infiltrated area with the highest microvessel density (hot spot).(4)

Fluorescent labeling and confocal microscopy

5TGM1 CCM was incubated with 1µM DiO (green fluorescence) (V-22887, Invitrogen, Thermo Fisher Scientific) for 15 minutes at room temperature prior to EV isolation by ExoQuick as described above. As a negative control, we added DiO to 10x concentrated serum-free medium, then followed the same steps of EV isolation as for 5TGM1 CCM. MC3T3-E1 cellular nuclei were stained using Hoechst staining (10µg/ml) and membranes were colored with Rhodamine Wheat Germ Agglutinin (Victor Laboratories, CA, USA) (25 µg/ml). After incubation of DiO-labeled exosomes with MC3T3-E1 cells for 24h, images of sEV uptake were acquired with a confocal laser scanning microscope LSM 710, using ZEN software (Carl Zeiss).

Collagen C-terminal Telopeptide concentration

Serum was collected from mice for measurement of circulating collagen type I degradation product, using the Mouse CTX/Collagen C-Terminal telopeptide ELISA kit (LifeSpan BioSciences, Inc., Seattle, WA, USA) according to manufacturer’s instructions. Samples were diluted 1:10. Absorbance was read at 450 nm using the BioRad iMark™ Microplate Absorbance reader (BioRad laboratories, Temse, Belgium).

Design of the animal treatment experiment

A power analysis was performed (Bonferroni correction, power: 0.8; effect size 1.6 and alpha error 0.0083) to determine the number of mice necessary in each group (n=10). Randomization occurred by allocating mice blindly to the different experimental groups. In the GW4869 group, three mice were excluded from analysis: one mouse died from myeloma before sacrifice and two mice were excluded based on a negative protein electrophoresis, indicating that there was no tumor inoculation.

Supplemental figure 1

A. Relative changes in trabecular bone volume, induced by 5TGM1 sEVs compared to 5TGM1 inoculation

B. Viability of 5TGM1 cells and differentiated RAW264.7 cells (osteoclasts) after treatment with 10 µM GW4869 for 24h.

C. Measurement of microvessel density (MVD) by counting CD31+ cells on BM sections

D. Verification of effects of 5TGM1 sEVs isolated by Optiprep Gradient ultracentrifugation on viability of MC3T3-E1

References:

1. Van Deun J et al. The impact of disparate isolation methods for extracellular vesicles on downstream RNA profiling. J Extracell Vesicles. 2014;3(1):1–14.

2. Heusschen R et al. SRC kinase inhibition with saracatinib limits the development of osteolytic bone disease in multiple myeloma. Oncotarget. 2016;7(21):30712–29.

3. De Bruyne E et al. IGF-1 suppresses Bim expression in multiple myeloma via epigenetic and posttranslational mechanisms. Blood. 2010;115(12):2430–40.

4. De Raeve HR, Vermeulen PB, Vanderkerken K, Harris AL, Van Marck E. Microvessel density, endothelial-cell proliferation and carbonic anhydrase IX expression in haematological malignancies, bone-marrow metastases and monoclonal gammopathy of undetermined significance. Virchows Arch. 2004;445(1):27–35.
