# Supplementary figures and images for: Exosomes play a role in multiple myeloma bone disease and tumor development by targeting osteoclasts and osteoblasts
Source: Blood Cancer J. 2018 Nov 8;8(11):105. doi: 10.1038/s41408-018-0139-7 (PMC6224554; doi:10.1038/s41408-018-0139-7)

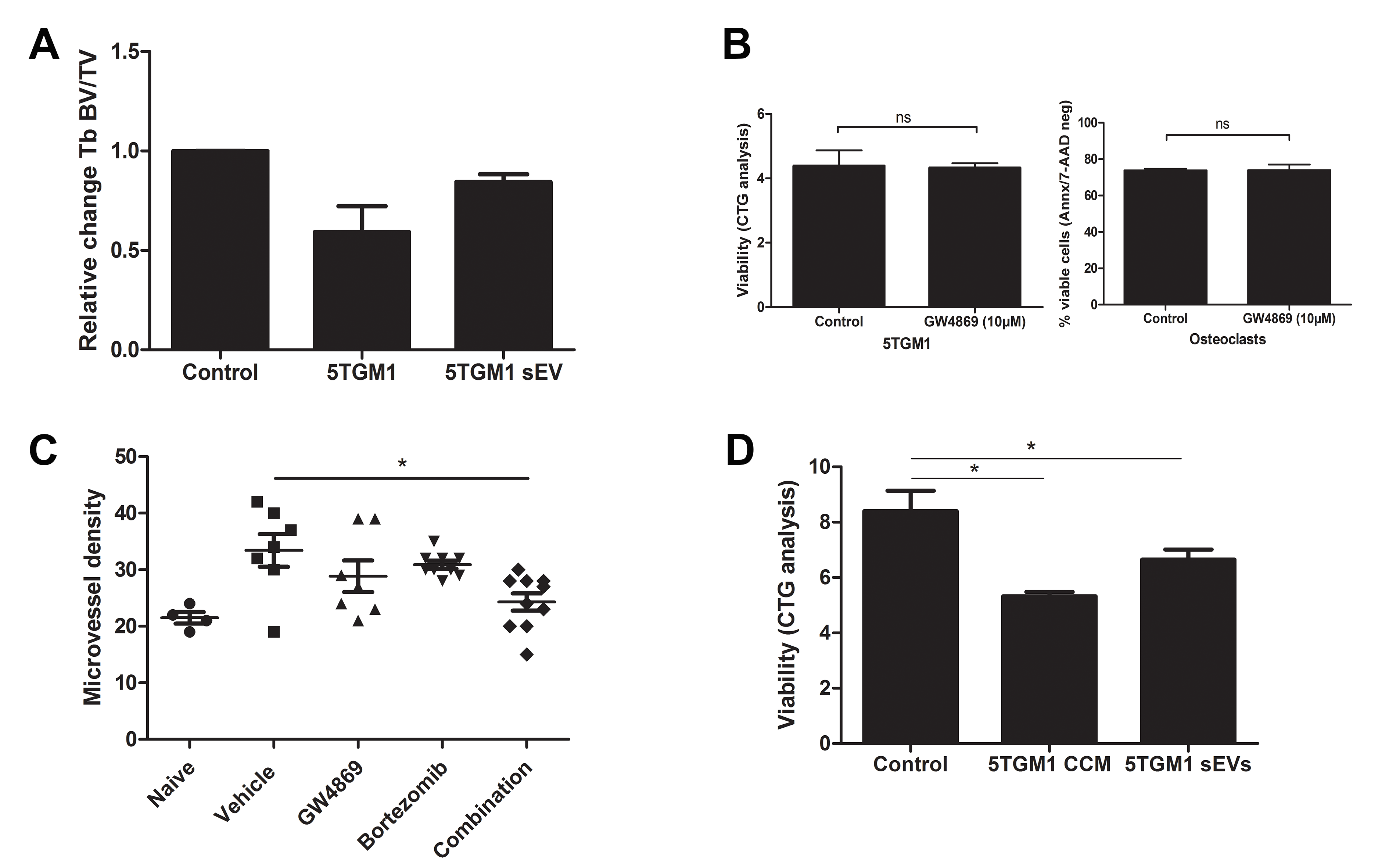

Supplement: Supplementary file 2 — supplemental figure 1 [file 41408_2018_139_MOESM2_ESM.tif]
